# Identification of 3,4-Dihydro-2*H*,6*H*-pyrimido[1,2-*c*][1,3]benzothiazin-6-imine Derivatives as Novel Selective Inhibitors of *Plasmodium falciparum* Dihydroorotate Dehydrogenase

**DOI:** 10.3390/ijms22137236

**Published:** 2021-07-05

**Authors:** Endah Dwi Hartuti, Takaya Sakura, Mohammed S. O. Tagod, Eri Yoshida, Xinying Wang, Kota Mochizuki, Rajib Acharjee, Yuichi Matsuo, Fuyuki Tokumasu, Mihoko Mori, Danang Waluyo, Kazuro Shiomi, Tomoyoshi Nozaki, Shinjiro Hamano, Tomoo Shiba, Kiyoshi Kita, Daniel Ken Inaoka

**Affiliations:** 1 Program for Nurturing Global Leaders in Tropical and Emerging Communicable Disease, Graduate School of Biomedical Science, Nagasaki University, Nagasaki 852-8523, Japan; endah.dwi08@yahoo.co.id (E.D.H.); rajibacharjee@cu.ac.bd (R.A.); shinjiro@nagasaki-u.ac.jp (S.H.); 2 Department of Parasitology, Institute of Tropical Medicine (NEKKEN), Nagasaki University, Nagasaki 852-8523, Japan; 3 Laboratory for Biotechnology, Agency for the Assessment and Application of Technology, South Tangerang 15314, Indonesia; danang.waluyo@bppt.go.id; 4 Department of Molecular Infection Dynamics, Institute of Tropical Medicine (NEKKEN), Nagasaki University, Nagasaki 852-8523, Japan; takaya.sakura@nagasaki-u.ac.jp (T.S.); erioy@nagasaki-u.ac.jp (E.Y.); 5 School of Tropical Medicine and Global Health, Nagasaki University, Nagasaki 852-8523, Japan; tagod9@hotmail.com (M.S.O.T.); wangxinying1229@hotmail.com (X.W.); ymatsuo@kumamoto-u.ac.jp (Y.M.); kitak@nagasaki-u.ac.jp (K.K.); 6 Department of Biomedical Chemistry, Graduate School of Medicine, The University of Tokyo, Tokyo 113-0033, Japan; nozaki@m.u-tokyo.ac.jp; 7 Department of Immunogenetics, Institute of Tropical Medicine (NEKKEN), Nagasaki University, Nagasaki 852-8523, Japan; a7m_kota@yahoo.co.jp; 8 Departement of Zoology, University of Chittagong, Chittagong 4331, Bangladesh; 9 Graduate School of Life Science, Kumamoto University, Kumamoto 860-0862, Japan; 10 Department of Cellular Architecture Studies, Institute of Tropical Medicine (NEKKEN), Nagasaki University, Nagasaki 852-8523, Japan; ftokumasu@nagasaki-u.ac.jp; 11 Biological Resource Center, NITE, Kisarazu, Chiba 292-0818, Japan; mori-mihoko@nite.go.jp; 12 Graduate School of Infection Control Sciences, Kitasato University, Tokyo 108-0072, Japan; shiomi@lisci.kitasato-u.ac.jp; 13 The Joint Usage/Research Center on Tropical Disease, Institute of Tropical Medicine (NEKKEN), Nagasaki University, Nagasaki 852-8523, Japan; 14 Department of Applied Biology, Graduate School of Science Technology, Kyoto Institute of Technology, Matsugasaki, Sakyo-ku, Kyoto 606-8585, Japan; tshiba@kit.ac.jp; 15 Department of Host-Defense Biochemistry, Institute of Tropical Medicine (NEKKEN), Nagasaki University, Nagasaki 852-8523, Japan

**Keywords:** *Plasmodium falciparum*, dihydroorotate dehydrogenase, inhibitor screening, ubiquinone, antimalarial drug

## Abstract

*Plasmodium falciparum*’s resistance to available antimalarial drugs highlights the need for the development of novel drugs. Pyrimidine de novo biosynthesis is a validated drug target for the prevention and treatment of malaria infection. *P. falciparum* dihydroorotate dehydrogenase (PfDHODH) catalyzes the oxidation of dihydroorotate to orotate and utilize ubiquinone as an electron acceptor in the fourth step of pyrimidine de novo biosynthesis. PfDHODH is targeted by the inhibitor DSM265, which binds to a hydrophobic pocket located at the N-terminus where ubiquinone binds, which is known to be structurally divergent from the mammalian orthologue. In this study, we screened 40,400 compounds from the Kyoto University chemical library against recombinant PfDHODH. These studies led to the identification of 3,4-dihydro-2*H*,6*H*-pyrimido[1,2-*c*][1,3]benzothiazin-6-imine and its derivatives as a new class of PfDHODH inhibitor. Moreover, the hit compounds identified in this study are selective for PfDHODH without inhibition of the human enzymes. Finally, this new scaffold of PfDHODH inhibitors showed growth inhibition activity against *P. falciparum* 3D7 with low toxicity to three human cell lines, providing a new starting point for antimalarial drug development.

## 1. Introduction

Significant morbidity and mortality caused by protozoan parasitic infection pose serious threats to global health. Among parasitic diseases, malaria is the most devastating, with approximately 409,000 deaths reported during 2019, 67% (274,000) of whom were children under 5 years old from sub-Saharan Africa [1,2]. Great effort has been spent to control malaria; however, the emergence of parasites resistant to practically all antimalarial drugs hampers the control and elimination of malaria. Therefore, the development of new antimalarial drugs is needed.

The majority of malaria cases reported are caused by *Plasmodium falciparum*. This parasite has a complex life cycle involving intermediary (human) and definitive (mosquitoes) hosts. Once a human is infected, the sporozoites migrate to the liver, invading hepatocyte (liver stage) and differentiating into schizont-containing hepatic merozoites. The hepatic schizonts then burst and release merozoites, which then infect red blood cells. During the blood stage, parasites undergo consecutive asexual development to ring, trophozoite, and schizont stages. Mature schizonts release merozoites into the bloodstream and initiate another replication cycle. A small fraction of parasites infecting erythrocytes undergo sexual development into female and male gametocytes, which are transmitted by mosquitoes during a blood meal.

Phenotypic and target-based screenings are the two approaches to obtain active compounds, the first step towards drug development. Artemisinin and its derivatives are one successful example of phenotypic screening and were introduced in 1972 [3] as antimalarial agents with an excellent safety profile. Artemisinin-based combination therapies (ACTs) are recommended by the World Health Organization (WHO) as the first-line treatment of uncomplicated *P. falciparum* malaria [4]. Because of the emergence of parasites resistant to ACTs, new antimalarial agents are being developed, such as spiroindolone KAE 609 (cipargamin), ozonides (e.g., OZ439), and imidazolopiperazines (KAF 156) [5,6,7], which are now in the development pipeline of the Medicine for Malaria Venture [8].

It is important to note that all the antimalarial drugs in clinical use were developed by phenotypic screening against the asexual blood stages, and only a few drugs are suggested to be active against the liver or mosquito stages. A new generation of antimalarial compounds targeting proteins essential in multiple stages is now under development. So far, several targets that have been chemically and/or genetically validated have been reported, such as, proteasome, phosphatidylinositol 4-kinase (PI4K), mitochondrial electron transport chain (ETC), and pyrimidine de novo biosynthesis [9,10].

The pyrimidine de novo biosynthesis pathway is an attractive antimalarial drug target. Humans can acquire pyrimidines through both de novo and salvage pathways, while the genes necessary for the salvage pathway are not present in *P. falciparum* [11]. Thus, *P. falciparum* is entirely dependent on the de novo pathway [12,13] for the supply of cellular pyrimidine. In addition, the pyrimidine de novo pathway is connected to the ETC at the level of ubiquinone through a reaction catalyzed by dihydroorotate (DHO) dehydrogenase (DHODH), the fourth and rate-limiting step of this pathway. DHODH is classified into two families (1 and 2) based on substrate/cofactor dependence and subcellular localization. Family 1 DHODHs are cytosolic enzymes, depending on the electron acceptor, and are subdivided into Families 1A and 1B. Family 1A DHODHs are homodimeric enzymes and share a single binding site for the substrate (DHO) and electron acceptor (fumarate) [14]. Family 1B DHODHs are heterotetrameric enzymes with long-range electron transfer from the flavin mononucleotide (FMN)-containing subunit (PyrDB) to an adjacent subunit (PyrK) containing an iron–sulfur cluster [2Fe-2S], flavin adenine dinucleotide (FAD), and an electron acceptor (NAD^+^) [15,16,17,18]. DHODHs belonging to Family 2 are membrane-bound enzymes that transfer the electrons from DHO to respiratory quinones, such as, ubiquinone, menaquinone, or rhodoquinone [19,20]. Family 2 enzymes are found in the plasma membrane of gram-negative bacteria and the mitochondrial inner membrane of eukaryotes.

Human DHODH (HsDHODH) is a drug target for the treatment of autoimmune diseases such as rheumatoid arthritis and psoriasis [21,22,23]. In general, inhibitors of bacterial, human, and plasmodial DHODHs share no cross-sensitivity [24,25,26,27]. Even between DHODHs from closely related apicomplexan parasites, such as *P. falciparum* (PfDHODH) and *Eimeria tenella* (EtDHODH), no cross-sensitivity has been observed [28]. However, a certain degree of cross-sensitivity is seen between EtDHODH and HsDHODH for two particular groups of inhibitors that are derived from ferulenol and ascofuranone [28]. Although the overall structure is shared between Family 2 DHODHs, the structure of the quinone binding site is diverse [28]. Such differences in the inhibitor binding site have been explored to design species-selective inhibitors, such as DSM265, a specific inhibitor of PfDHODH [29] with remarkable activity against the blood and liver stages of *P. falciparum* but not *P. vivax* [30]. Although DSM265 showed promising results in a Phase 2a study by showing rapid parasite clearance after single-dose treatment [30], it is no longer included in the Medicine for Malaria Venture (MMV) portfolio [31,32], possibly due to side effects (clinical trial identifier: NCT02450578). Thus, the identification of PfDHODH inhibitors with different chemical structures and better safety profiles than DSM265 is needed. The search for new PfDHODH inhibitors has involved natural sources, including plants and microorganisms [33].

**Figure 1:**
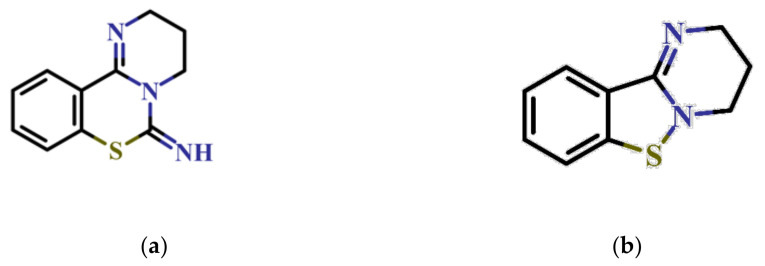
Structures of 3,4-dihydro-2*H*,6*H*-pyrimido[1,2-*c*][1,3]benzothiazin-6-imine (**a**) and 3,4-dihydro-2*H*-benzo[4,5]isothiazolo[2,3-*a*]pyrimidine (**b**).

In this study, we screened 40,400 compounds from the Kyoto University chemical library [34] to discover novel PfDHODH inhibitors that are active against the asexual blood-stage parasite. Here, we report the new chemical scaffold 3,4-dihydro-2*H*,6*H*-pyrimido[1,2-*c*][1,3]benzothiazin-6-imine (DPBI, Figure 1a) and its analog 3,4-dihydro-2*H*-benzo[4,5]isothiazolo[2,3-*a*]pyrimidine (DBIP, Figure 1b) as well as their derivatives as selective PfDHODH inhibitors at a sub-micromolar to low-micromolar order with low cytotoxicity to human cells.

## 2. Results

### 2.1. Identification of DPBI and DBIP Derivatives as PfDHODH Inhibitors

**Figure 2:**
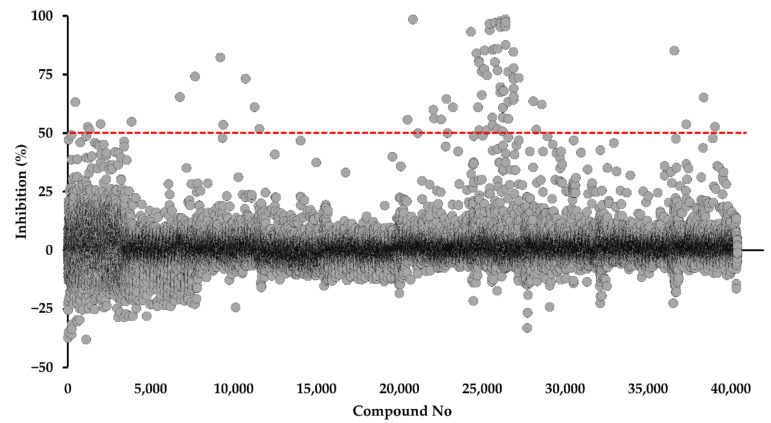
Screening of 40,400 compounds from Kyoto University chemical library against PfDHODH. The screening was performed at a final concentration of 4.5 μM. The dashed line represents the 50% inhibition threshold for the selection of hits.

His6-SUMO-tagged PfDHODH was successfully expressed and purified from the *Escherichia coli* membrane. After digestion by SUMO protease, we purified the tag-free enzyme with a specific activity of 22.3 µmol/min/mg (*K*_cat_ = 17.5 s^−1^), which was used for screening 40,400 compounds from the Kyoto University chemical library. The quality of our screening was evaluated by calculating the following parameters: Z’-factor (0.88 ± 0.09), signal window (SW, 49.2 ± 20.3), signal-to-noise ratio (S/N = 85.0 ± 26.0), signal-to-background (S/B = 115.3 ± 28.6), and negative coefficient of variation (CV = 2.38 ± 1.85%), all of which were excellent. After the initial screening at 4.5 µM, we identified 43 compounds that meet the hit criteria (>50% inhibition), for a hit rate of 0.11% (Figure 2). According to the library policy, the chemical structures are disclosed only for the hits. After analysis of their chemical structures, we classified the hits as derivatives of DPBI or DBIP, which are listed in Table 1, Table 2, Table 3 and Table 4.

### 2.2. Structure–Activity Relationship of DPBI Derivatives

Of the 43 hits obtained in this study, 27 compounds were DPBI derivatives (Table 1 and Table 2 and Figure S1). The introduction of a 2-napthyl group at the R_1_-position of DPBI showed the highest inhibition (**1**, IC_50_ = 0.65 µM) against PfDHODH. Substitution at R_1_ to other 2-membered rings, such as Compounds **2** to **7,** did not drastically affect the IC_50_s against PfDHODH (IC_50_ ranging from 0.70 to 1.3 µM) as shown in Table 1. Similarly, substitution with a styrene group at R_1_ (**8**, IC_50_ = 1.07 µM) or a phenyl group at R_2_ (**9**, IC_50_ = 0.76 µM) maintained the activity towards PfDHODH. Dual substitutions with benzothiophene and tert-butyl groups at R_1_ and R_3_ (**10**, IC_50_ = 3.84 µM), respectively, resulted in a 6-fold increase in the IC_50_ compared to **1** (Table 1). Changing the backbone structure from DPBI to 2,3,4,6-tetrahydro-7-thia-1,5-diazatetraphen-6-imine (**11**) resulted in an increased IC_50_ to 6.42 µM relative to **1**. Substitutions of the 2-naphtyl group of 1 to a phenyl group (**12**) had little effect on IC_50_ (Table 2). Additional substitutions at R_1_, R_2_, or R_3_ from Compound **12** showed modest increases in the IC_50_ ranging from 0.96 to 3.89 µM against PfDHODH (Compounds **13** to **27**; Table 2).

### 2.3. Structure–Activity Relationship of DBIP Derivatives

As a result of screening, 16 derivatives of BIP were included in the hits (Table 3 and Table 4 and Figure S2). BIP derivatives listed in Table 3 having acetamido (**28**) or trifluoromethyl (29) substitutions at R_1_ showed the highest IC_50_s of 18.5 and 9.71 µM against PfDHODH, respectively. Other BIP derivatives with substitutions at R_1_ (**30** to **35**) or R_2_ (**36**) inhibited PfDHODH activity stronger than **28** (Table 3). Moreover, polar substitutions on the benzene group of **30** at R_1_ (**37** to **39**), R_2_ (**40** and **41**), or R_3_ (**42** and **43**) lowered the IC_50_ compared to **30** (Table 4).

### 2.4. Counter-Assays Against HsDHODH and Mammalian Complex I, II, and III Activities

To investigate whether or not the hits obtained in this study also inhibit HsDHODH, a counter-assay was conducted using purified recombinant HsDHODH. As a result, none of the hits inhibited HsDHODH (Table 1, Table 2, Table 3 and Table 4). Except for orotate derivatives, all known Family 2 DHODH inhibitors bind to the ubiquinone binding site. To test the hypothesis that the inhibitors of PfDHODH obtained in this study also bind to the ubiquinone binding site of mammalian respiratory complexes I, II, and III, inhibition assays against the activities of complexes I–III and II–III were conducted. As shown in Table 1, Table 2, Table 3 and Table 4, except for **2**, which mildly inhibited mammalian complex I–III activity (IC_50_ = 13.9 µM), the remaining hits did not inhibit the activities of mammalian complexes I-III and II-III, at least in the range tested (0.0007 to 22.7 µM) in this study.

### 2.5. Antimalarial Activity of PfDHODH Inhibitors from the Kyoto University Chemical Library

Next, in vitro antimalarial activities of the 43 hits were evaluated against *P. falciparum* 3D7 based on PfLDH/diaphorase assays. A total of 31 compounds displayed EC_50_s below 10 µM, while the other 12 compounds did not inhibit parasite growth. From the compounds exhibiting antimalarial activity, 19 compounds showed an EC_50_ between 1 and 10 µM, and 12 compounds were below 1 µM (Table 1, Table 2, Table 3 and Table 4).

### 2.6. Cytotoxicity Assay of Mammalian Cells

The cytotoxicities of the hits were assessed against three different human cell lines: HDF, PANC-1, and DLD-1. A total of 5 DBIP derivative compounds, **36**, **37**, **40**, **42**, and **43**, showed weak cytotoxicity (Table S1) against the normal cell line (HDF), with EC_50_ values of 8.38, 8.43, 9.16, 9.77, and 9.44 µM, respectively (Figure S3). None of the tested compounds affected the growth of the two cancer cell lines PANC-1 and DLD-1 (Table S1).

### 2.7. Confirmation Assay against the Transgenic Parasites

To test whether the expression of yDHODH in *P. falciparum* 3D7 could confer resistance to the PfDHODH inhibitors identified in this study, relative parasite growth at 10 µM was analyzed. As shown in Table 1, Table 2, Table 3 and Table 4, Pf3D7-yDHODH parasite showed reduced susceptibility to several compounds, **6**, **13**, **14**, **19**, **21**, **30**, and **43**. Some hits inhibited the growth of both wild-type and Pf3D7-yDHODH, suggesting the presence of off-target(s) in *P. falciparum* (Table 1, Table 2, Table 3 and Table 4 and Figure S4).

## 3. Discussion

The current drugs used to treat malaria were developed by classical phenotypic screening targeting the asexual blood stage. For effective malaria eradication, antimalarial drugs targeting multiple life cycle stages are desired [35,36], such as the gametocyte (transmission-blocking activity) and/or sporozoite (chemoprophylaxis) stages, in addition to the intraerythrocytic stage. In that sense, pyrimidine de novo biosynthesis is an attractive drug target because of its essentiality in at least the asexual and sporozoite stages.

To discover selective PfDHODH inhibitors with a novel scaffold, we screened 40,400 compounds from Kyoto University chemical library. This library is open-access and composed of many original compounds and diverse scaffolds (https://www.pharm.kyoto-u.ac.jp/pgcg/library.html) (accessed on 10 May 2021). Our efforts led to the identification of a new class of PfDHODH inhibitors that share DPBI and DBIP moieties. Those derivatives have previously been reported to inhibit the proliferation of human immunodeficiency virus (HIV) [37,38,39,40,41,42], hepatitis C virus [42,43], and herpes simplex virus [41]. The 43 PfDHODH inhibitors identified in this study showed no inhibition against the human enzyme or mammalian mitochondrial complexes I–III and II–III, except for 2, which weakly inhibited complex I–III activity (Table 1). This result suggests that those compounds have little or no effect on the ETC of host mitochondria. The majority of those inhibitors were active against *P. falciparum* 3D7 and displayed low toxicity to mammalian normal and cancer cell lines.

Family 1 DHODHs are insensitive to Family 2 DHODH inhibitors because they lack the ubiquinone binding site [44]. Hence, transgenic *P. falciparum*-expressing Family 1A DHODHs, such as yDHODH, are known to be resistant to PfDHODH inhibitors [45,46]. Such strains have been used to probe the mechanism of action of new antimalarials that target PfDHODH. In this study, the antimalarial activity and mechanism of action were confirmed for **6**, **13**, **14**, **19**, **21**, **30**, and **43**, which showed growth inhibition against 3D7 but were inactive against 3D7-yDHODH (Table 1, Table 2 and Table 4). Several derivatives were able to inhibit the growth of both 3D7 and 3D7-yDHODH strains, which were more pronounced in DBIP than DPBI derivatives, indicating the presence of additional target(s) (Table 1, Table 2, Table 3 and Table 4, Figure S4).

In this study, we have identified PfDHODH inhibitors with new chemical scaffolds that inhibited the growth of the *P. falciparum* 3D7 strain. Further activity profiling, such as activity against multi-drug resistant strains (Dd2 or K strains), liver-stage parasites, as well as in vivo models (*P. berghei*) will be required for the future development of the compounds described in this study.

## 4. Materials and Methods

### 4.1. Expression and Purification of Recombinant PfDHODH

The codon-optimized PfDHODH gene, encoding amino acid residues 158 to 569 fused to a His6-SUMO tag in its N-terminus, was constructed and optimized for expression in *E. coli*, as recently reported [33]. Briefly, the *E. coli* BL21 Star (DE3) strain, harboring pETSUMO/PfDHODH, was selected in Luria–Bertani agar plates supplemented with 50 µg/mL kanamycin (Sigma). A single colony was inoculated onto 360 mL of LB medium containing the same antibiotic (pre-culture) and cultured at 37 °C for 16 h with shaking at 200 rpm. The pre-culture (60 mL) was transferred to 600 mL of Terrific-Broth media supplemented with 50 µg/mL kanamycin in an Ultra Yield^®®^ flask (Thomson), and then cultured at 37 °C with vigorous shaking at 200 rpm (a total of 6 flasks). Protein expression was induced by 25 µM isopropyl β-d-1-thiogalactopyranoside (IPTG, Sigma) when the optical density at 600 nm reached 0.6–0.8, and then cultured for 16 h at 20 °C.

The subsequent purification steps were performed at 4 °C. Cells were harvested by centrifugation at 5000× *g* for 10 min, and then resuspended in lysis buffer containing 50 mM HEPES-NaOH (Dojindo) pH 7.6, 50 mM NaCl (Wako), 5 mM imidazole (Wako), 20% (*v/v*) glycerol (Wako), and 0.25 mM phenylmethylsulfonyl fluoride (PMSF, Wako). Suspended cells were disrupted by a French press (Ohtake) at 180 MPa, followed by centrifugation at 30,000× *g* for 20 min to discard unbroken cells and debris. Triton X-100 (Roche) was mixed with a supernatant to a final concentration of 1% (*w/v*), stirred for 30 min, and then further centrifuged at 40,000 × g for 90 min. The supernatant was mixed with 3 mL of pre-equilibrated nickel-nitriloacetic acid (Ni-NTA, Qiagen) resin and kept overnight, rotating at 10 rpm. The protein–resin mixture was centrifuged at 1500× *g* for 15 min and the resin was suspended in a minimal volume of supernatant and loaded into a gravity-flow column. Next, the column was sequentially washed with 60 mL of buffer A (50 mM HEPES-NaOH pH 7.6, 300 mM NaCl, 10% (*v/v*) glycerol, 0.2 mM orotate), 60 mL of buffer A containing 0.05% (*w/v*) polyoxyethylene(9)dodecyl ether (C_12_E_9_, Anatrace), and followed by 60 mL of buffer A containing 0.05% C_12_E_9_ and 20 mM imidazole. The bound protein was eluted with buffer A containing 0.05% C_12_E_9_ and 300 mM imidazole. The eluted fraction was concentrated using a centrifugal filter unit (Amicon Ultra-50, 50 kDa molecular weight cut-off (MWCO), Millipore) at 3500× *g* until the final volume reached 0.5 mL. The concentrated protein plus detergent solution was diluted with buffer A containing 0.05% (*w/v*) C_12_E_9_ until the imidazole concentration was reduced to 75 mM.

The His6-SUMO tag was cleaved by SUMO protease at a 1:50 ratio relative to PfDHODH to a final volume of 40 mL in cleavage buffer (50 mM Tris-HCl pH 8.0, 0.05% (*w/v*) C_12_E_9_, and 0.2 mM orotate) and incubated for 14 h. Equilibrated Ni-NTA resin was added into the cleavage mix and then incubated for 2.5 h at 4 °C, followed by loading onto a gravity-flow column. The flow-through was collected and concentrated as described above. An equal volume of cold glycerol was added to the concentrated protein, FMN was added to a final concentration of 200 µM, and it was stored at −30 °C until use.

### 4.2. Screening of the Kyoto University chemical Library

The screening of 40,400 compounds was conducted in 384-well plates by adapting the end-point assay reported before [47]. First, 1 µL of 200 µM compound was transferred into 384-well plates using a Benchtop Multi-Pipetter EDR-384SR (Biotech Co., Ltd., Tokyo, Japan). The same volume of dimethyl sulfoxide (DMSO, Dojindo) was added to columns 1 and 2 as negative controls (0% inhibition), while 18% (*w/v*) sodium dodecyl sulfate (SDS, Wako) was added to columns 23 and 24 as positive controls (100% inhibition). Next, 38 µL of assay mix (100 mM HEPES-NaOH pH 7.5, 5% (*v/v*) glycerol, 150 mM NaCl, 0.05% (*v/v*) Triton X-100, 15 µM decylubiquinone (dUQ, Sigma), 120 µM 2,6-dichlorophenolindophenol (DCIP, Sigma), and 20 nM PfDHODH) [28] were dispensed into all wells and mixed at 600 rpm for 1 min by a MixMate^®®^ (Eppendorf). The reaction was started by the addition of 5 µL of 1.8 mM L-DHO (Sigma) as the substrate and mixed as described above for 20 s. The absorbance at 600 nm was recorded using a SpectraMax^®®^ Paradigm^®®^ Multi-Mode Microplate Reader (Molecular Devices) before (t0) and after 20 min (t20) incubation at room temperature. The readings at t0 were subtracted from t20, and PfDHODH inhibition was calculated as the inhibition relative to the negative and positive controls in a single-point assay. Hits were defined as compounds inhibiting more than 50% of PfDHODH activity at 4.5 µM. The quality of the screening system was evaluated by calculating statistical parameters: Z′-factor, S/N, S/B, SW, and CV, as previously reported [48,49]. The IC_50_ values of the hit compounds were determined using the same assay system containing serial dilutions of each compound in triplicates (22.7, 6.8, 2.27, 0.68, 0.227, 0.068, 0.023, 0.007, 0.002, 0.0007 µM) using GraphPad Prism 8.0 software (GraphPad Software Inc., San Diego, CA, USA).

### 4.3. HsDHODH Assay

Selected hit compounds from PfDHODH screening were tested for potential inhibition against HsDHODH at 4.5 µM. The HsDHODH used in this study was purified as previously reported [50]. The HsDHODH assay was performed similarly to that for PfDHODH, but in an assay mix consisting of 50 mM Tris-HCl pH 8.0, 0.1% (*v/v*) Triton X-100, 2 mM potassium cyanide (KCN, Wako), 60 µM dUQ, and 120 µM DCIP, with the reaction started by the addition of 200 µM L-DHO (final concentration) in triplicate. For this assay, DMSO was used as a negative control and 10 µM of lapachol was used as a positive control (100% inhibition). The relative inhibition was calculated as described above.

### 4.4. Mammalian Complex I, II, and III Activity Assays

Bovine heart mitochondria were prepared as previously described [50,51,52]. The NADH-cytochrome *c* reductase (complexes I–III) and succinate-cytochrome *c* reductase (complexes II–III) activities were assayed in 384-well plates following an established protocol [53,54,55,56] with small modifications. Complex I–III activity was assayed in a reaction mix containing 50 mM phosphate buffer pH 7.4, 2 mM KCN, 200 µM cytochrome *c* (cytochrome *c* from horse heart, Nacalai tesque), and 15 µg/mL mitochondria. Plates containing 1 µL serial dilutions of PfDHODH inhibitors were prepared and mixed with 38 µL of the reaction mix, as described above. The reaction was started by the addition of 5 µL of 2.6 mM NADH, and the assay was performed as described for PfDHODH, but at 550 nm and with 15 min of incubation. Complex II–III activity was assayed similarly to complex I–III activity but with 100 µM cytochrome *c* and 25 µg/mL mitochondria, and the reaction was started by 10 mM succinate. For both assays, DMSO and 5 µM ascochlorin were used as negative and positive controls, respectively. Assays were performed in triplicate.

### 4.5. In vitro Antimalarial Assay

This assay was conducted using human red blood cells (RBC) following the guidelines of the ethics committee of Nagasaki University (permission no. 19). Human RBC was obtained from the Japanese Red Cross Society. The *P. falciparum* 3D7 strain was cultured in 2% hematocrit type O human RBC in RPMI-1640 medium (Gibco) supplemented with 23.8 mM sodium bicarbonate (Wako), 50 mg/L hypoxanthine (Wako), 25 mg/L gentamycin (Sigma), and 0.5% (*w/v*) AlbuMAX^®®^ II (Gibco) under a 5% O_2_, 5% CO_2_, and 90% N_2_ atmosphere at 37 °C. Parasites were synchronized with 5% (*w/v*) D-sorbitol (Wako).

Synchronized ring-stage parasites at 0.3% parasitemia and 2% hematocrit were cultured in a 384-well plate at 25 µL with test compounds at a final concentration in the range of 0.0003 to 10 µM with a fixed DMSO concentration of 0.4% (*v/v*). Parasite growth was monitored by the diaphorase-coupled *P. falciparum* lactate dehydrogenase (PfLDH) assay, as previously described [57,58]. After 72 h, 70 µL of the assay solution (150 mM lithium L-lactate (Wako), 0.05 mg/mL 3-acetylpyridine adenine dinucleotide (Oriental Yeast Co., Ltd., Tokyo, Japan), 0.2 mg/mL nitro blue tetrazolium (Wako), 1 unit/mL diaphorase, and 100 mM Tris-HCl pH 8.0) were mixed with the parasite culture and incubated at room temperature for 40 min, and the generated nitro blue formazan was measured at 650 nm using a SpectraMax^®®^ Paradigm^®®^ Multi-Mode Microplate Reader. The first and second columns contained 0.4% (*v/v*) DMSO as a negative control, and the 23rd and 24th columns had a mixture of 1 µM atovaquone and artemisinin as a positive control. The EC_50_ was calculated using GraphPad Prism 8.0 software from the mean of quadruplets values.

### 4.6. Cytotoxicity Assays of Mammalian Cells

Human dermal fibroblast cells (HDF, Zenbio, Inc., Durham, NC, USA) were cultured in Dulbecco’s Modified Eagle Medium/Nutrient Mixture F-12 medium (DMEM/F12, Gibco), human pancreatic carcinoma cells (PANC-1, DS Pharma Biomedical, Co., Ltd.) were cultured in DMEM (Gibco), and human colorectal adenocarcinoma cells (DLD-1, Taiho Pharmaceutical Company) were cultured in RPMI-1640 medium (FUJIFILM Wako Pure Chemicals). The culture mediums were supplemented with 10% (*v/v*) heat-inactivated fetal bovine serum (Gibco) and the cells were maintained at 37 °C under 5% CO_2_.

HDF, PANC-1, and DLD-1 were seeded at 2.5 × 10^4^ cells/well on a 96-well plate and incubated at 37 °C under 5% CO_2_ overnight. The cells were then washed with PBS and the medium was replaced with 99 µL of fresh media. Then, 1 µL of the compounds or DMSO was added to the respective wells. As controls, wells containing 1% (*v/v*) DMSO in column 1 and wells containing only the culture medium in column 12 were used as the negative and positive controls, respectively. After 48 h of incubation, the cells were washed with PBS, and 99 µL of fresh media was replaced, followed by the addition of 10 µL of Cell Counting Kit-8 (Dojindo) to each well. After 3 h of incubation, the absorbance was measured at 450 nm using a SpectraMax^®®^ Paradigm^®®^ Multi-Mode microplate Reader. The calculation of cell viability in the test well was based on the absorbance of control wells according to the manufacturer’s protocol. The mean of quadruplet values was used to calculate the CC_50_ using GraphPad Prism 8.0 software.

### 4.7. Generation of Transgenic Parasite

The *P. falciparum* 3D7 strain expressing the cytosolic yeast DHODH (3D7-yDHODH) was prepared as follows. pHHyDHODH-GFP plasmid (Kerafast, Boston, MA, USA) was transfected to the *P. falciparum* 3D7 strain following the established transfection method [59]. Briefly, 50 µg of plasmid dissolved in cytomix solution (120 mM KCl, 0.15 mM CaCl_2_, 10 mM K_2_HPO_4_/KH_2_PO_4_ pH 7.6, 25 mM HEPES-KOH pH 7.6, 2 mM ethylene glycol-bis(β-aminoethyl ether)-*N*,*N*,*N*′,*N*′-tetraacetic acid (EGTA, Dojindo), and 5 mM magnesium chloride (Wako)) was transfected into the red blood cells by the Gene Pulser Xcell (Bio-Rad). Percoll–sorbitol synchronized parasites were mixed with red blood cells containing plasmid and maintained under 5 nM WR99210 for 2 weeks. The growth of resistant parasites against DSM265 and atovaquone, as PfDHODH and complex III inhibitors respectively, was confirmed by PfLDH/diaphorase assay. A total of 10 µM PfDHODH inhibitors were tested against wild-type 3D7 and 3D7-yDHODH following the method described above in triplicate. The significance of difference between the inhibition of the two parasite strains was evaluated by Student’s *t*-test (Figure S4).

## 5. Conclusions

In this work, 40,400 compounds from the Kyoto University chemical library were screened against PfDHODH. Several quality control parameters were calculated in every assay plate, indicating excellent performance of our screening. The reproducibility of inhibition was further confirmed by the dose-response assay and IC_50_ determination. We successfully identified new classes of PfDHODH inhibitor harboring DPBI and DBIP moieties with growth inhibition activity against the asexual stage of *P. falciparum* 3D7. As DHODH does not seem to be essential in sexual stages, future studies will focus on the activity of our compounds against the liver stage parasite. Moreover, the compounds identified in this study are selective for PfDHODH and displayed low toxicity in several human cell lines, providing a new starting point for future antimalarial drug development.

## Figures and Tables

**Table 1:** Antimalarial activities of DPBI derivatives.

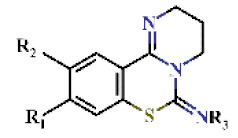									
**Cpd**	**Substituents**			**IC_50_ (µM)**				**EC_50_ (µM)**	**Growth at 10 µM (%)**
	**R_1_**	**R_2_**	**R_3_**	**DHODH**		**Mammalian Mitochondrial**		**Pf3D7**	**Pf3D7-yDHODH**
				**Pf**	**Hs**	**CI–III**	**CII–III**		
**1**	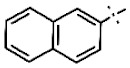	H	H	0.65 ± 0.05	>4.5	>22.7	>22.7	0.43 ± 0.18	−0.62 ± 0.07
**2**	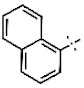	H	H	0.85 ± 0.05	>4.5	13.9 ± 2.14	>22.7	>10	nd
**3**	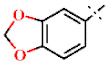	H	H	1.18 ± 0.04	>4.5	>22.7	>22.7	1.16 ± 0.02	−0.23 ± 0.01
**4**	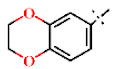	H	H	0.97 ± 0.08	>4.5	>22.7	>22.7	1.07 ± 0.05	24.0 ± 5.76
**5**	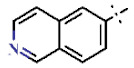	H	H	0.96 ± 0.09	>4.5	>22.7	>22.7	1.13 ± 0.05	26.0 ± 8.27
**6**	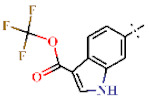	H	H	0.70 ± 0.04	>4.5	>22.7	>22.7	0.37 ± 0.05	104.4 ± 0.11
**7**	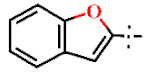	H	H	1.30 ± 0.05	>4.5	>22.7	>22.7	0.34 ± 0.01	36.2 ± 1.01
**8**	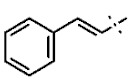	H	H	1.07 ± 0.05	>4.5	>22.7	>22.7	0.63 ± 0.25	0.12 ± 0.21
**9**	H	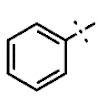	H	0.76 ± 0.08	>4.5	>22.7	>22.7	>10	nd
**10**	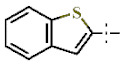	H	(CH_3_)_3_	3.84 ± 0.09	>4.5	>22.7	>22.7	2.77 ± 0.28	−0.48 ± 0.07
**11**	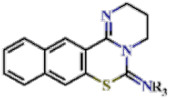		H	6.42 ± 0.09	>4.5	>22.7	>22.7	>10	nd

Several derivatives that were not active against *P. falciparum* 3D7 were not assayed against transgenic 3D7-yDHODH. The IC_50_ value represents the concentration of each compound that inhibits PfDHODH, HsDHODH, CI-III, and CII-III activities by 50%. The EC_50_ value represents the concentration of each compound that inhibits Pf3D7 growth by 50%. Values and errors are averages and standard deviations of at least n = 3. Compounds listed in bold were showing EC_50_ < 1 µM against Pf3D7. The chemical structures are colored according to the CPK scheme (oxygen atom = red; nitrogen atom = blue; sulfur atom = deep yellow; and fluorine atom = light green). nd = not determined.

**Table 2:** Antimalarial activities of Compound **12** derivatives.

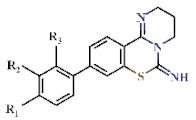									
**Cpd**	**Substituents**			**IC**_**50**_ ** (µM)**				**EC**_**50**_ ** (µM)**	**Growth at 10 µM (%)**
	**R**_**1**_	R_**2**_	R_**3**_	**DHODH**		**Mammalian Mitochondrial**		**Pf3D7**	**Pf3D7-** **yDHODH**
				**Pf**	**Hs**	**CI-III**	**CII-III**		
**12**	H	H	H	0.68 ± 0.05	>4.5	>22.7	>22.7	1.15 ± 0.07	−0.22 ± 0.12
**13**	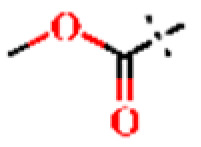	H	H	1.22 ± 0.05	>4.5	>22.7	>22.7	1.06 ± 0.01	70.5 ± 0.73
**14**	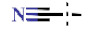	H	H	3.89 ± 0.23	>4.5	>22.7	>22.7	1.21 ± 0.04	105 ± 0.47
**15**	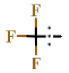	H	H	2.31 ± 0.11	>4.5	>22.7	>22.7	1.00 ± 0.05	−0.38 ± 0.17
**16**	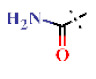	H	H	1.82 ± 0.08	>4.5	>22.7	>22.7	>10	nd
**17**	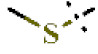	H	H	0.96 ± 0.07	>4.5	>22.7	>22.7	1.62 ± 0.56	−0.50 ± 0.01
**18**	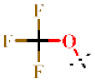	H	H	1.43 ± 0.06	>4.5	>22.7	>22.7	0.37 ± 0.13	2.54 ± 0.42
**19**	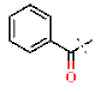	H	H	1.07 ± 0.05	>4.5	>22.7	>22.7	0.51 ± 0.14	68.6 ± 0.43
**20**	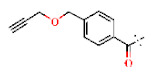	H	H	1.21 ± 0.06	>4.5	>22.7	>22.7	0.38 ± 0.08	0.45 ± 0.74
**21**	H	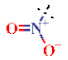	H	3.68 ± 0.31	>4.5	>22.7	>22.7	0.85 ± 0.12	103 ± 0.67
**22**	H	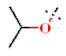	H	1.56 ± 0.05	>4.5	>22.7	>22.7	0.90 ± 0.44	−0.06 ± 0.24
**23**	H	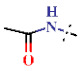	H	1.85 ± 0.19	>4.5	>22.7	>22.7	>10	nd
**24**	H	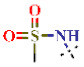	H	1.94 ± 0.07	>4.5	>22.7	>22.7	>10	nd
**25**	H	C_2_H_5_	H	1.52 ± 0.08	>4.5	>22.7	>22.7	1.77 ± 0.83	0.01 ± 0.15
**26**	H	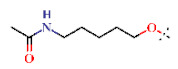	H	3.49 ± 0.21	>4.5	>22.7	>22.7	>10	nd
**27**	H	H	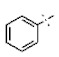	3.30 ± 0.10	>4.5	>22.7	>22.7	0.39 ± 0.05	0.25 ± 0.04

Several derivatives that were not active against *P. falciparum* 3D7 were not assayed against transgenic 3D7-yDHODH. The IC_50_ value represents the concentration of each compound that inhibits PfDHODH, HsDHODH, CI-III, and CII-III activities by 50%. The EC_50_ value represents the concentration of each compound that inhibits Pf3D7 growth by 50%. Values and errors are averages and standard deviations of at least n = 3. Compounds listed in bold were showing EC_50_ < 1 µM against Pf3D7. The chemical structures color codes are described in Table 1. Nd = not determined.

**Table 3:** Antimalarial activity of DBIP derivatives.

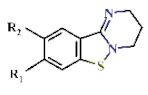								
**Cpd**	**Substituents**		**IC**_**50**_ ** (µM)**				**EC**_**50**_ ** (µM)**	**Growth at 10 µM (%)**
	**R**_**1**_	R_**2**_	**DHODH**		**Mammalian** **Mitochondrial**		**Pf3D7**	**Pf3D7-** **yDHODH**
			**Pf**	**Hs**	**CI–III**	**CII–III**		
**28**	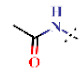	H	18.5 ± 0.40	>4.5	>22.7	>22.7	>10	nd
**29**	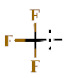	H	9.71 ± 0.29	>4.5	>22.7	>22.7	>10	nd
**30**	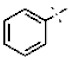	H	4.26 ± 0.12	>4.5	>22.7	>22.7	9.67 ± 5.09	63.6 ± 5.87
**31**	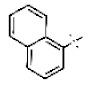	H	1.10 ± 0.08	>4.5	>22.7	>22.7	0.33 ± 0.01	0 ± 0.07
**32**	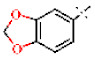	H	1.29 ± 0.09	>4.5	>22.7	>22.7	1.85 ± 0.53	0.11 ± 0.03
**33**	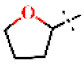	H	1.53 ± 0.04	>4.5	>22.7	>22.7	3.73 ± 0.88	44.1 ± 26.2
**34**	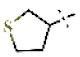	H	0.94 ± 0.05	>4.5	>22.7	>22.7	1.64 ± 0.35	−0.1 ± 0.28
**35**	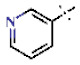	H	1.48 ± 0.07	>4.5	>22.7	>22.7	>10	nd
**36**	H	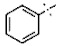	1.51 ± 0.04	>4.5	>22.7	>22.7	4.16 ± 0.09	32.5 ± 10.1

Several derivatives that were not active against *P. falciparum* 3D7 were not assayed against transgenic 3D7-yDHODH. The IC_50_ value represents the concentration of each compound that inhibits PfDHODH, HsDHODH, CI-III, and CII-III activities by 50%. The EC_50_ value represents the concentration of each compound that inhibits Pf3D7 growth by 50%. Values and errors are averages and standard deviations of at least n = 3. Compounds listed in bold were showing EC_50_ < 1 µM against Pf3D7. The chemical structures color codes are described in Table 1. Nd = not determined.

**Table 4:** Antimalarial activity of Compound 30 derivatives.

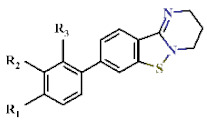									
**Cpd**	**Substituents**			**IC**_**50**_ ** (µM)**				**EC**_**50**_ ** (µM)**	**Growth at 10 µM (%)**
	**R**_**1**_	R_**2**_	R_**3**_	**DHODH**		**Mammalian Mitochondrial**		**Pf3D7**	**Pf3D7-** **yDHODH**
				**Pf**	**Hs**	**CI–III**	**CII–III**		
**37**	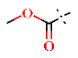	H	H	1.15 ± 0.07	>4.5	>22.7	>22.7	2.78 ± 0.27	−0.18 ± 0.05
**38**	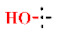	H	H	1.81 ± 0.01	>4.5	>22.7	>22.7	>10	nd
**39**	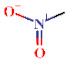	H	H	1.30 ± 0.04	>4.5	>22.7	>22.7	1.14 ± 0.06	1.48 ± 0.14
**40**	H	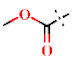	H	1.43 ± 0.02	>4.5	>22.7	>22.7	1.72 ± 0.48	−0.09 ± 0.08
**41**	H	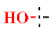	H	1.44 ± 0.05	>4.5	>22.7	>22.7	>10	nd
**42**	H	H	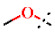	1.18 ± 0.05	>4.5	>22.7	>22.7	0.54 ± 0.22	0.04 ± 0.15
**43**	H	H	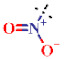	1.15 ± 0.06	>4.5	>22.7	>22.7	1.11 ± 0.08	31.1 ± 2.38

Several derivatives that were not active against *P. falciparum* 3D7 were not assayed against transgenic 3D7-yDHODH. The IC_50_ value represents the concentration of each compound that inhibits PfDHODH, HsDHODH, CI-III, and CII-III activities by 50%. The EC_50_ value represents the concentration of each compound that inhibits Pf3D7 growth by 50%. Values and errors are averages and standard deviations of at least n = 3. Compounds listed in bold were showing EC_50_ < 1 µM against Pf3D7. The chemical structures color codes are described in Table 1. Nd = not determined.

## Data Availability

Not applicable.

## Supplementary Materials

The following are available online at https://www.mdpi.com/article/10.3390/ijms22137236/s1. Figure S1: The inhibitory activity of DPBI derivatives against PfDHODH and their chemical structure; Figure S2: The inhibitory activity of DBIP derivatives against PfDHODH and their chemical structures; Figure S3: Normalized normal cell line (HDF) viability after 48 h; Figure S4: Antimalarial activity of PfDHODH inhibitors against *P. falciparum* 3D7 and 3D7-yDHODH at 10 µM. Data are presented as the mean ± SD (n = 3). Significance of difference between the two parasites was tested by Student’s *t*-test. The asterisk marks a significant difference at the following levels: * = *p* < 0.05, ** = *p* < 0.01, *** = *p* < 0.001, **** = *p* < 0.0001; Table S1: Cytotoxicity assays of DPBI and DBIP derivatives against human cell lines (HDF, PANC-1, and DLD-1).
