# Supplementary material for: Identification of 3,4-Dihydro-2H,6H-pyrimido[1,2-c][1,3]benzothiazin-6-imine Derivatives as Novel Selective Inhibitors of Plasmodium falciparum Dihydroorotate Dehydrogenase
Source: Int J Mol Sci. 2021 Jul 5;22(13):7236. doi: 10.3390/ijms22137236 (PMC8268581; doi:10.3390/ijms22137236)
Supplement: Supplementary file 1 [file ijms-22-07236-s001.zip › ijms-1267010-supplementary.pdf]

Supplementary Materials

Table S1: Cytotoxicity assays of DPBI and DBIP derivatives against human cell lines (HDF, PANC-1, and DLD-1).

Compound	EC_{50} (μM)		
	HDF	PANC-1	DLD-1
1	>10	>10	>10
2	>10	>10	>10
3	>10	>10	>10
4	>10	>10	>10
5	>10	>10	>10
6	>10	>10	>10
7	>10	>10	>10
8	>10	>10	>10
9	>10	>10	>10
10	>10	>10	>10
11	>10	>10	>10
12	>10	>10	>10
13	>10	>10	>10
14	>10	>10	>10
15	>10	>10	>10
16	>10	>10	>10
17	>10	>10	>10
18	>10	>10	>10
19	>10	>10	>10
20	>10	>10	>10
21	>10	>10	>10
22	>10	>10	>10
23	>10	>10	>10
24	>10	>10	>10
25	>10	>10	>10
26	>10	>10	>10
27	>10	>10	>10
28	>10	>10	>10
29	>10	>10	>10
30	>10	>10	>10
31	>10	>10	>10
32	>10	>10	>10
33	>10	>10	>10
34	>10	>10	>10
35	>10	>10	>10
36	8.38 ± 0.46	>10	>10
37	8.43 ±1.9	>10	>10
38	>10	>10	>10
39	>10	>10	>10
40	9.16 ± 1.1	>10	>10
41	>10	>10	>10
42	9.77 ± 0.28	>10	>10
43	9.44 ± 2.30	>10	>10

Data are presented as the mean ± SD (n=3)

Figure S1: The inhibitory activity of DPBI derivatives against PfDHODH and their chemical structures.

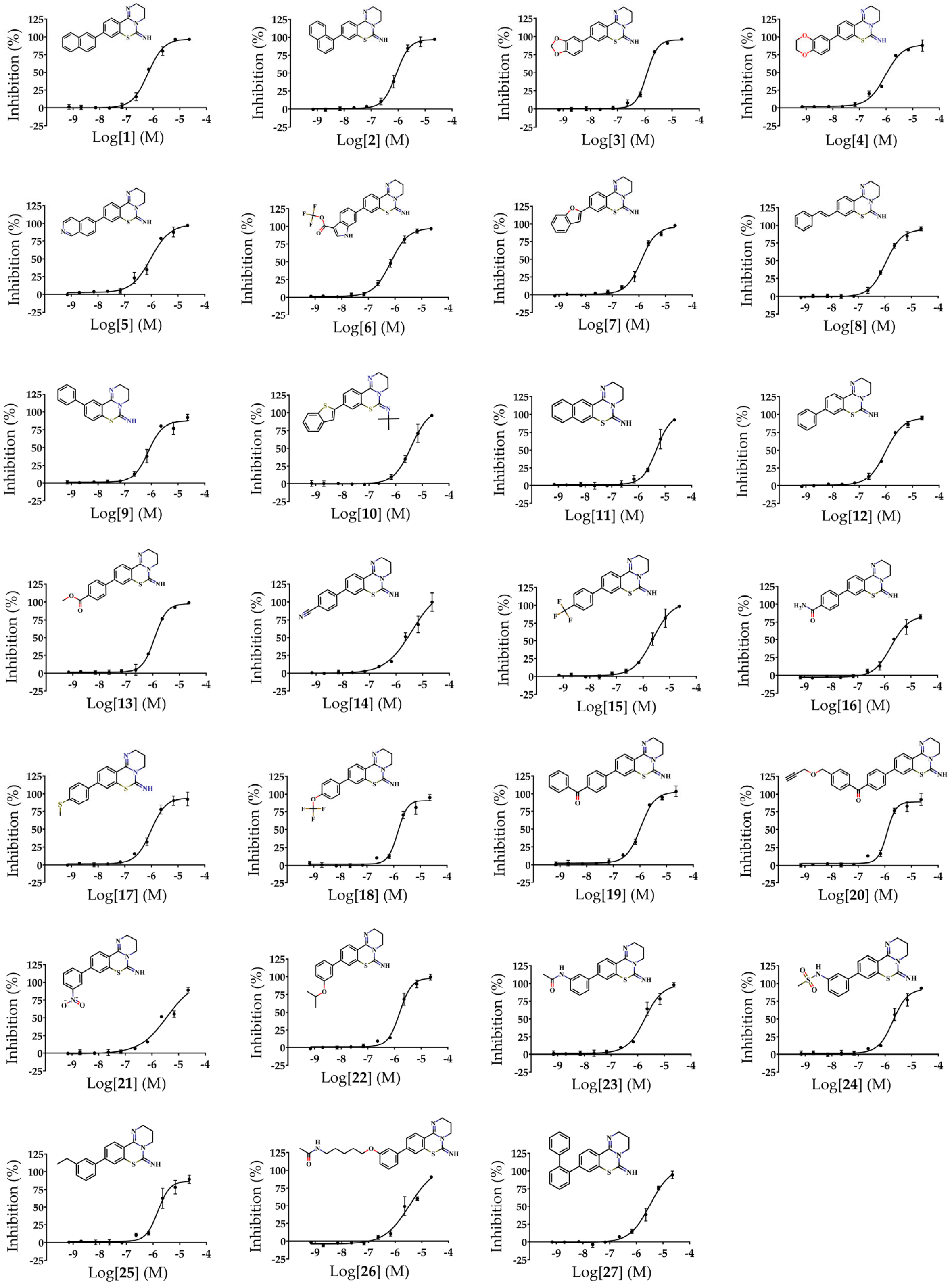

Figure S2: The inhibitory activity of DBIP derivatives against PfDHODH and their chemical structures.

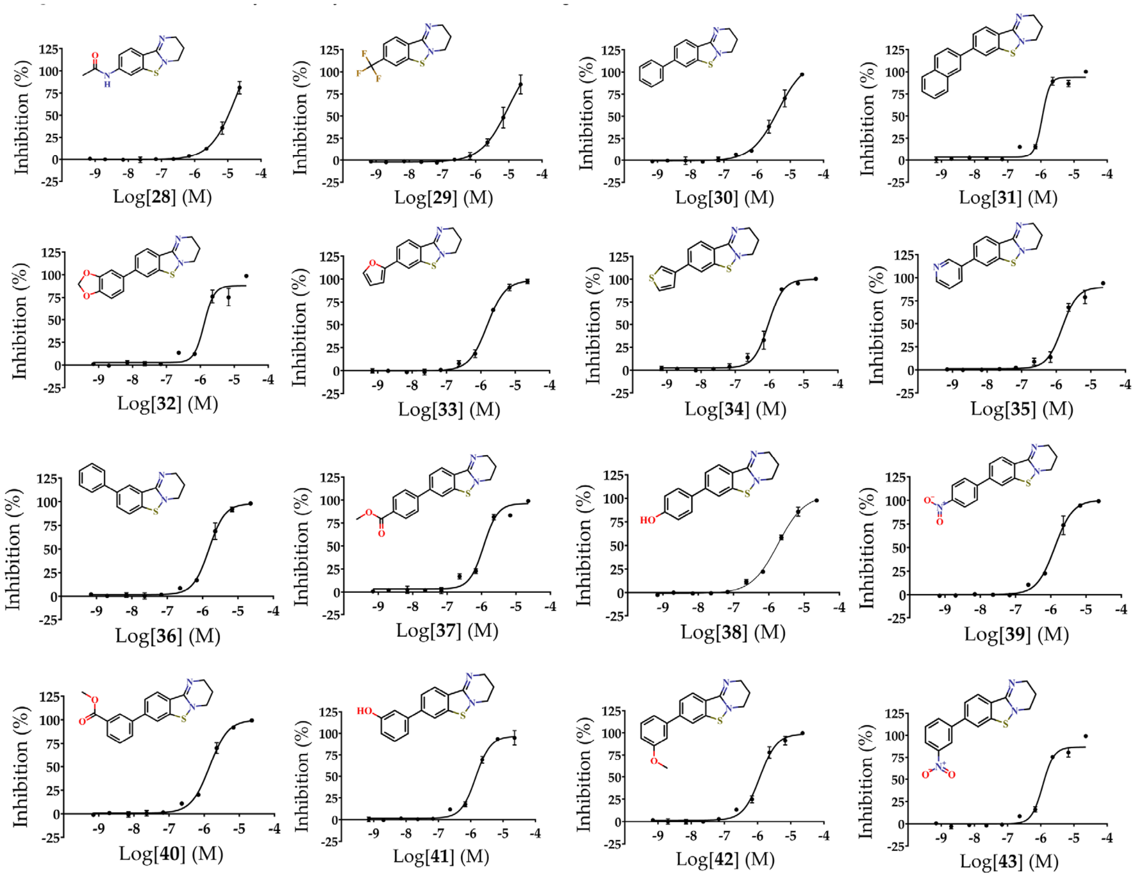

Figure S3: Normalized normal cell line (HDF) viability after 48 h.

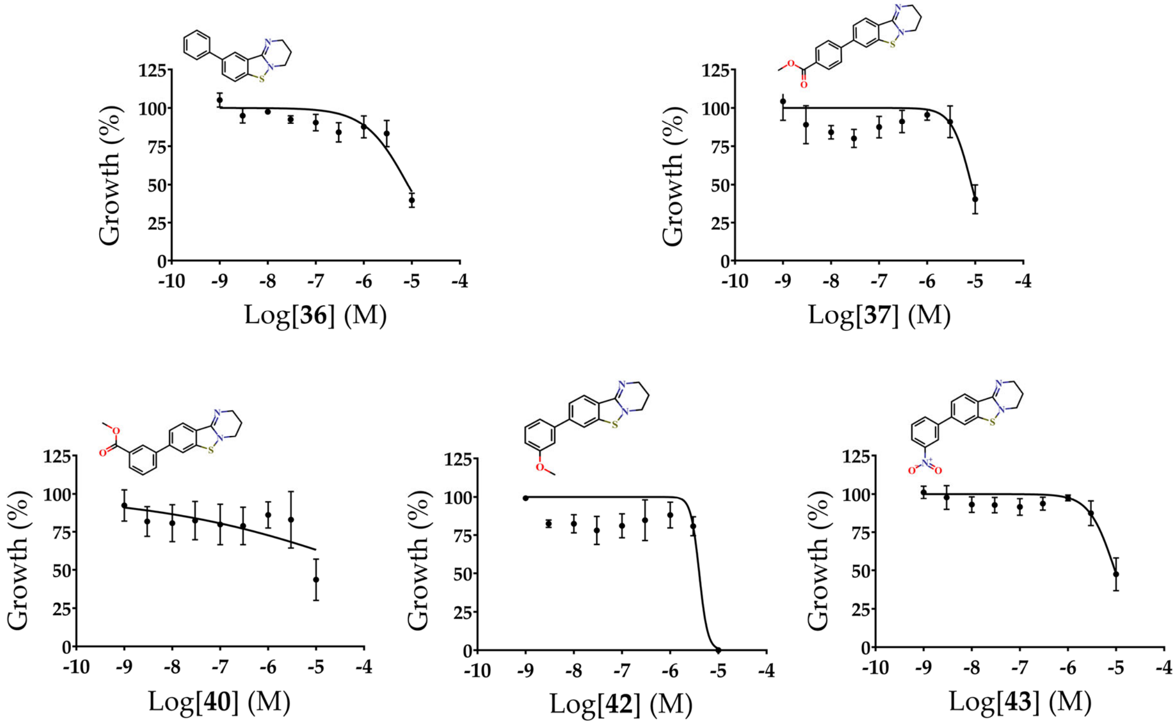

Figure S4: Antimalarial activity of PfDHODH inhibitors against *P. falciparum* 3D7 and 3D7-yDHODH at 10 μM.

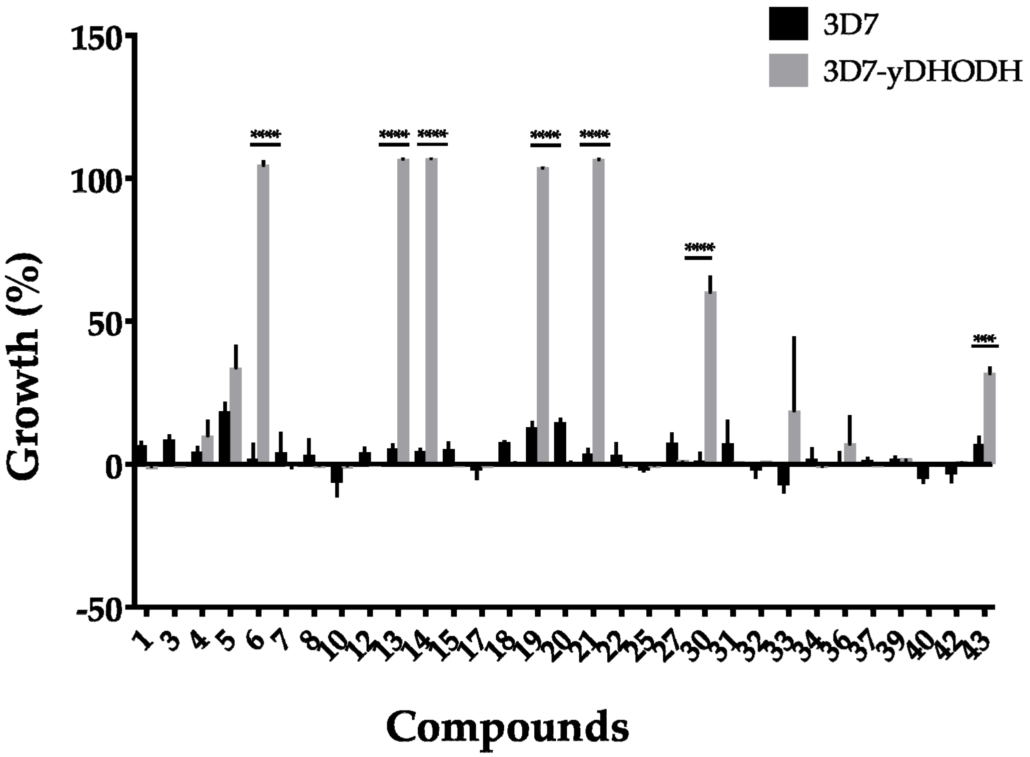

Data are presented as the mean ± SD (n=3). Significance in difference between two parasites were tested by Student's *t* test. The asterisk marks a significant difference at the level of: * = p < 0.05, ** = p < 0.01, *** = p < 0.001, **** = p < 0.0001.
